# Comprehensive morphological, immunohistochemical, and molecular characterization of congenital pulmonary airway malformation (CPAM)

**DOI:** 10.1007/s00428-025-04131-4

**Published:** 2025-06-06

**Authors:** S. Schweitzer, M. Stiller, M. Lacher, O. Aubert, S. Krämer, F. W. Hirsch, D. Gräfe, K. Hauptmann, A. Arnold, D. Horst, M. Chirica, F. Klauschen, U. Obeck, M. Boeschen, H. Bläker, M. von Laffert

**Affiliations:** 1 Institute of Pathology, Leipzig University, Liebigstraße 26, 04103 Leipzig, Germany; 2 Department of Pediatric Surgery, Leipzig University, Leipzig, Germany; 3 Department of Visceral, Transplantation, Thoracic, and Vascular Surgery, Leipzig University, Leipzig, Germany; 4 Department of Pediatric Radiology, Leipzig University, Leipzig, Germany; 5 Institute of Pathology, Charité–Universitätsmedizin Berlin,, Corporate Member Freie Universität Berlin and Humboldt-Universität zu Berlin, Berlin, Germany; 6 Institute of Pathology, Ludwig-Maximilians-Universität München, Munich, Germany

**Keywords:** Congenital pulmonary airway malformation, Comparison of mucinous and non-mucinous regions, *KRAS* mutation, *FGFR2* mutation, Mucinous cell clusters

## Abstract

Congenital pulmonary airway malformation (CPAM) is among the most common congenital lung disorders. Possible findings are foci of mucinous cell clusters (MCCs), harboring *KRAS* mutations. Studies combining morphology, immunohistochemistry (IHC), and molecular data are scarce. A total of. 46 CPAM lesions were assessed by conventional histomorphology. IHC was performed to analyze lung-specific markers, transcription factors, and epithelial markers reflecting the structural composition of the bronchial tree and alveolar system. Next-generation sequencing (NGS) was conducted to identify mutations and fusions. CPAM type 1 was found in 50%, type 2 in 22%, and type 3 in 6%. A mixed pattern of types 1 and 2 was observed in 22%. The epithelial lining was strongly positive for CK7 and TTF-1 in all samples. Expression for Napsin A, Surfactant A, p40, CK5/6, and CK20 varied within and between subtypes. P40 and CK5/6 were more enriched in type 1 compared to type 2. MCCs occurred in 17%. Mutations were found in 24% (9 × *KRAS*; 2 × *FGFR2*). Besides classical *KRAS* mutations (G12D, G12V), two cases showed a double-mutation pattern (G12D/G12V; G12D/*TP53*). In all MCC cases, the same mutation with comparable allele frequencies was found in mucinous and non-mucinous areas. No fusions were detected. The presence of mixed-type patterns supports the hypothesis that CPAM represents a continuum of developmental disturbances occurring at various stages of lung branching morphogenesis. This is further corroborated by the divergent protein expression patterns. Our study confirms recent findings that the same *KRAS* mutations occur in mucinous and non-mucinous areas. The identification of additional mutations, including potential co-mutations, underscores the need for further investigation into molecularly defined CPAM subtypes.

**Supplementary Information:**

The online version contains supplementary material available at https://doi.org/10.1007/s00428-025-04131-4.

## Introduction

Congenital pulmonary airway malformation (CPAM), formerly known as congenital cystic adenomatoid malformation (CCAM), is a cystic hamartomatous lesion of the lung [1, 2]. With an estimated incidence ranging from 1 in 7200 to 1 in 35,000 live births [3, 4], it is among the most common congenital pulmonary anomalies [2]. The Stocker classification (types 0–4) categorizes CPAMs based on macroscopic appearance and histopathological features [5, 6]. Types 0 and 4 are considered distinct entities; notably, type 4 is discussed as the cystic variant of pleuropulmonary blastoma [6, 7]. Types 1 to 3 are thought to originate from developmental disturbances during different stages of lung branching morphogenesis, particularly the pseudoglandular phase (5th to 17th week of gestation). However, the exact pathogenesis remains unclear [2, 7, 8].

Mucinous cell clusters (MCCs) have been identified in up to 83% of CPAM type 1 cases [9], but are less frequently observed in other subtypes [10, 11]. These clusters commonly harbor *KRAS* mutations [10, 12–15], raising concerns about their potential role as precursors to malignancy [10, 12, 15, 16]. Several case reports have documented progression from MCCs to mucinous adenocarcinoma [4, 13, 17–21]. In some cases, diagnosis of carcinoma has been based primarily on the presence of a *KRAS* mutation [19, 21], prompting the question of whether similar genetic alterations might also be present in the adjacent non-mucinous cystic epithelium. Recent molecular studies have compared MCCs with neighboring non-mucinous cystic epithelia. While Fakler et al. found no *KRAS*-mutations outside of MCCs [10], other studies—such as a case report by Koopman et al. [22] and larger analyses by Hermelijn et al. [12] and Nelson et al. [16]—identified identical *KRAS* mutations in both mucinous and non-mucinous regions. These findings highlight ongoing controversy regarding the molecular landscape of CPAM. Moreover, comprehensive immunohistochemical analyses of CPAMs—including comparisons of lung-specific markers, transcription factors, and epithelial markers reflecting the composition of the bronchial tree and alveolar system—are scarce. To address these gaps, we conducted a comparative analysis of 46 CPAM cases at the morphological, immunohistochemical, and molecular levels.

## Material and methods

### Patients and samples

We analyzed formalin-fixed, paraffin-embedded (FFPE) tissue from 46 CPAM lesions obtained from 44 patients across three centers (Leipzig: 27 cases; Munich: 12 cases; Berlin: 7 cases). The median age at the time of surgery was 6.8 months (range: 3 days to 40 years). Surgical intervention consisted of at least a lobectomy, performed between 2003 and 2024. Further details are provided in Supplementary Table 1.

### Conventional morphology

Where available, cyst dimensions were extracted from pathological reports, adhering to the established size criteria (> 2 cm for type 1; ≤ 2 cm for type 2). CPAM classification into types 1, 2, and 3 was based on the histopathological criteria originally described by Stocker [5, 6], with refinements from more recent literature [9, 23]. Type 1 was referred to as “wavy,” type 2 as “punched-out,” and type 3 as “microcystic” pattern. For a detailed histomorphological description and definition of these types—which may also serve as a basis for routine diagnostic practice—please refer to Table 1, based on the most recent literature.

**Table 1:** Detailed histological description and definition of different CPAM (congenital pulmonary airway malformation) types and of the distribution and localization of MCCs (mucinous cell clusters) according to Stocker [5, 6], with refinements from more recent literature [9, 23]

Morphological pattern	Histological definition
CPAM type 1 (“wavy”)	Epithelium of a bronchial type with possible epithelial complexity (papillary projections), irregularly shaped small cysts and bronchial structures (such as smooth muscle and cartilage) in the cyst wall Cysts are lined by ciliated cuboidal to stratified columnar epithelium with interspersed alveolar-type spaces Frequent transitions from thick cyst wall to adjacent alveolar wall Solid appearing features possible with presence of mucinous cell clusters (MCC, see below)
CPAM type 2 (“punched-out”)	Readily identifiable cysts to mildly malformed alveolar-type spaces Typically, round, lined by ciliated columnar, bronchiolar and/or ductal epithelium type with thin or absent layers of smooth muscle cells Cyst formations may be focal Complexity of epithelium is rare No or rare transitions from cystic spaces into adjacent alveolar-type spaces Striated skeletal muscle may occasionally be seen in the septa between cysts
CPAM type 3 (“microcystic”)	Solid or spongiform microcysts Predominantly small irregular airway spaces lined by ciliated to columnar epithelium Interspersed alveolar spaces with thin walls and flat pneumocytes are rare or absent Surrounding septa often appear thickened, with prominent mesenchyme and cuboidal epithelium Solid appearing features possible with presence of mucinous cell clusters (MCC, see below)
MCC	Distribution: **Unifocal** vs. **multifocal** Localization: **Intra-lesional** (inside the CPAM) vs. **extra-lesional** (outside the CPAM) Intra-lesional was further divided into: • **Intracystic:** In contact with the cystic epithelium, growing directly adjacent to or projecting into the cyst lumen • **Extracystic:** Without contact to the cystic epithelia, located outside the cyst epithelium or cyst lumen, within the stroma/tissue of the remaining CPAM

Mucinous cell clusters (MCCs) were measured in millimeters, and their distribution/localization was documented as further outlined in Table 1.

The histopathological evaluation included a total of 349 slides (mean: 7.6 slides per lesion; range: 1–36), all stained with Hematoxylin and Eosin (H&E). Additionally, periodic acid-Schiff (PAS) staining was performed on 98 slides (mean: 2.1 slides per lesion; range: 1–10).

### Immunohistochemistry

For immunohistochemical analysis (IHC), one representative FFPE block per CPAM lesion was selected, displaying the most prominent diagnostic features—including large cysts, characteristic epithelial patterns, and mucinous cell clusters (if present)—as summarized in Table 1. Staining encompassed lung-specific markers (Napsin A and Surfactant A), transcription factors (TTF-1, CDX2, and SATB2), and epithelial markers reflecting the composition of the bronchial tree and alveolar system (p40, CK5/6, CK7, and CK20).

Evaluation was scored as follows: negative (−): total absence of positively stained cells (0%); partial positive (+): incomplete, discontinuous staining of lower intensity in up to 40% of the epithelial cells; strong positive (+ +): predominantly continuous, intense staining in more than 40% of the epithelial cells. Staining was performed on the Ventana BenchMark Ultra (Roche, Basel, Switzerland) according to the manufacturer’s instructions (for further information, see Supplementary Table 2).

### Nucleic acid isolation and next-generation sequencing (NGS)

All 46 different CPAM types were separately analyzed by NGS on the DNA and RNA level. Guided by pre-evaluated H&E-stained reference slides, manual macrodissection was performed on paraffin-embedded tissue sections using a scalpel to selectively isolate mucinous and non-mucinous epithelial regions prior to DNA and RNA extraction. Special care was taken to prevent cross-contamination between the two compartments. Macrodissection was carried out on 46 CPAM cysts without mucinous cell clusters (MCCs) and on all 8 distinct CPAM cases with MCCs, resulting in a total of 54 separately processed samples for downstream NGS analysis on the DNA and RNA level. For targeted DNA sequencing, a custom QIAseq targeted DNA panel (Qiagen) covering the partial or complete coding region of 40 genes was used. Fusion gene analysis was performed by a custom QIAseq targeted RNAscan panel covering the breakpoints of 110 genes. Sequencing was performed on the Miseq instrument (Illumina). For a detailed description and gene list, see SupplementaryMethods 1 and Supplementary Tables 3 and 4.

### Statistics

Statistical analyses were performed using RStudio (version 4.3.2) [24]. Kruskal–Wallis tests were used to analyze differences between types 1, 2, and mixed for the variables cyst size, Napsin A, Surfactant A, p40, CK5/6, and CK20. Significant differences were further analyzed using Dunn’s tests as post hoc tests. Given the limited number of type 3 cases, this subtype was not included in the statistical analyses. To compare (previously named markers) between *KRAS*-mutated vs. *KRAS* wild-type samples, Mann–Whitney *U* tests were performed. *P*-values were adjusted using the false discovery rate (FDR) method (*q*-values) and considered significant for *q* < 0.05.

## Results

### Conventional morphology

**Fig. 1:**
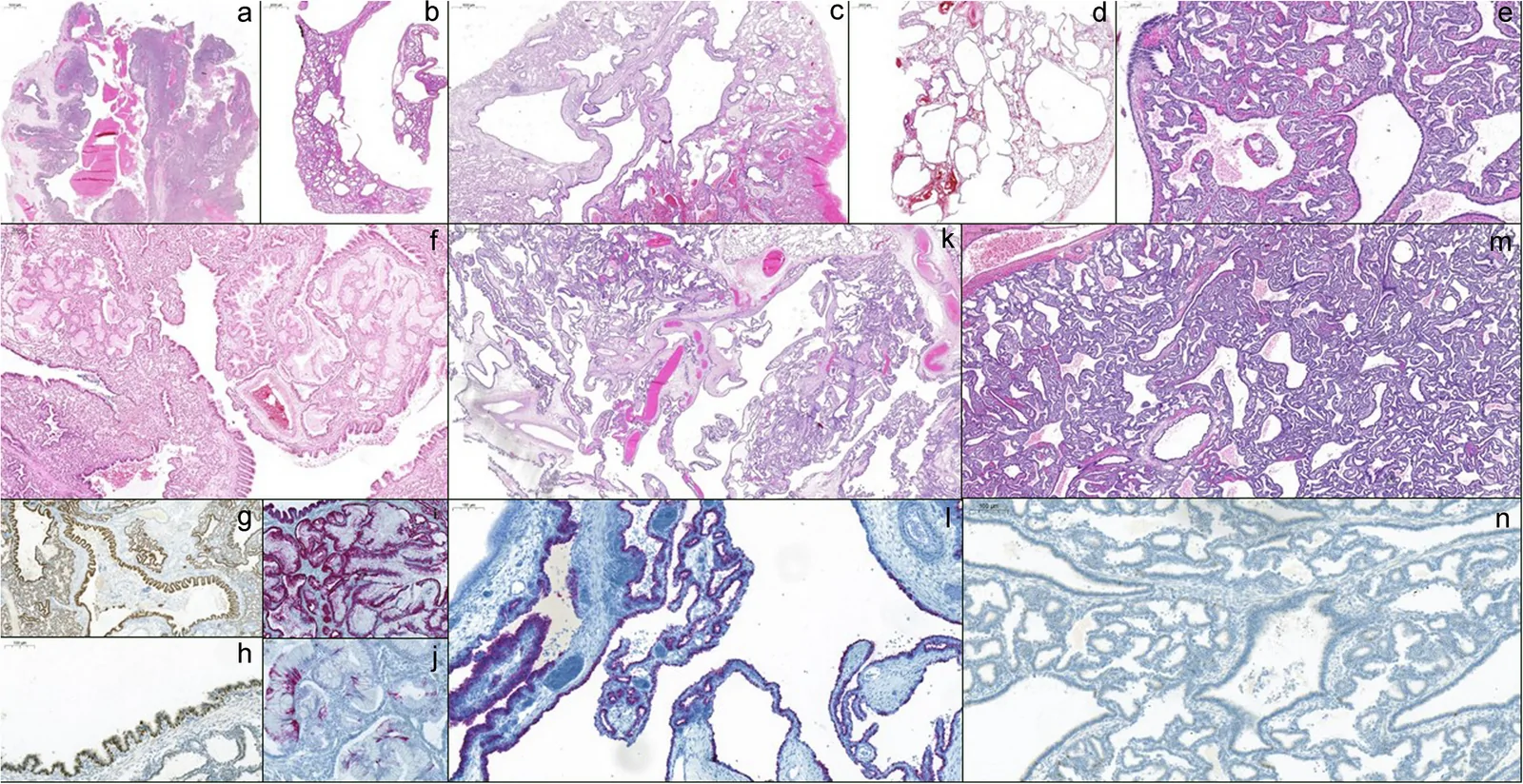
The different CPAM subtypes and selected immunohistochemical aspects (**a–n**): Type 1 exhibited a “wavy” pattern with papillary projections (**a**); mixed type 1 + 2 showed both “wavy” and “punched-out” patterns (**b, c**); type 2 cysts showed a “punched-out” pattern only, with a back-to-back configuration (**d**); type 3 a “microcystic” pattern (**e**). A CPAM type 1 with prominent mucinous cell clusters (MCCs) is shown in (**f**), the cyst is positive for TTF1 (**g**) and p40 (**h**), the MCCs for CK7 (**i**) and CK20 (**j**). In (**k**), a CPAM type 1 without MCC is depicted, strongly positive for Napsin A (**l**). In (**m**), the “microcystic” pattern of type 3 is shown; here, p40 is only focally positive (**n**)

A total of 50% (23/46) of the samples were classified as stocker type 1 (showing a “wavy” pattern), 22% (10/46) as type 2 (“punched-out” pattern), and 6% (3/46) as type 3 (“microcystic” pattern). 22% (10/46) showed mixed histological features encompassing patterns of type 1 and 2. Here, cysts lined by pseudostratified ciliated cells and those resembling dilated bronchioles were found within one lesion (Fig. 1).

A total of 43% (20/46) of all samples showed cyst sizes ≤ 2 cm, 52% (24/46) > 2 cm; for two samples, no size was documented. All type 3 lesions showed spongiform cystic areas (diameters: 4.5 cm, 5.5 cm, and 11.0 cm) with small microcysts with a few millimeters of diameter only (Table 2). No statistically significant differences were found between CPAM types (1, 2, mixed) and cyst size concerning the 2 cm cut-off (*p* = 0.16; *q* = 0.24).

**Table 2:** Detailed comparison of different CPAM types and cyst size based on the 2 cm cut-off. For type 1 lesions, a tendency above 2 cm, and for type 2 and mixed lesions, below 2 cm can be seen; however, the difference is not statistically significant. For one type 2 lesion (*) and one mixed type (**), cyst size was not documented in the pathological report

CPAM type	Cyst size ≤ 2 cm	Cyst size > 2 cm	Total
1	9/23 (39%)	14/23 (61%)	23/23 (100%)
2	6/10 (60%)	3/10 (30%)	9*/10 (90%)
Mixed	6/10 (60%)	3/10 (30%)	9**/10 (90%)
3	3/3 (100%)	0/3 (0%)	3/3 (100%)

MCCs occurred in 17% (8/46) of all samples, with a frequency of 30% (7/23) in type 1 and 33% (1/3) in type 3 (Fig. 1). Sizes ranged from 0.5 to 1.6 mm (mean 0.9 mm). All eight cases showed intra-lesional arrangement of MCCs, seven with a multifocal and one with unifocal pattern. The unifocal cluster (size 0.5 mm) was initially not visible by H&E section but became apparent within deeper slide sections by means of PAS reaction. Six of the multifocal MCCs showed both intra- and (predominantly) extracystic distribution. One case had an intracystic pattern only. No extra-lesional MCCs were found in the surrounding regular lung tissue.

### Immunohistochemistry

The cyst epithelia were strongly positive for CK7 and TTF-1 in all CPAMs. Partial or strong positivity for Napsin A, Surfactant A, p40, CK5/6, and CK20 was found in 61% (28/46), 57% (26/46), 100% (46/46), 83% (38/46), and 46% (21/46), respectively. Intensity of the expression patterns varied within and between subtypes. All MCCs showed combined positivity for CK7 and CK20 only. All samples were negative for CDX2 and SATB2. For a detailed description of IHC results, see Table 3 and Fig. 1.

**Table 3:** Detailed expression patterns and distributions (%) of Napsin A, Surfactant A, TTF-1, p40, CK5/6, CK7, and CK20 in the cyst epithelia regarding the different CPAM subtypes. Differences between types were tested using Kruskal–Wallis tests (*p*-values) and adjusted using the false discovery rate method (*q*-values); (*the positivity of p40 in type 3 lesions was focal only)

IHC	CPAM type (*n*)	Negative (−)	Partial positive (+)	Strong positive (+ +)	*P*-value	*Q*-value
**Napsin A**	**1** (23)	35% (8/23)	48% (11/23)	17% (4/23)	0.37	0.37
	**Mixed** (10)	30% (3/10)	70% (7/10)	0% (0/10)		
	**2** (10)	60% (6/10)	30% (3/10)	10% (1/10)		
	**3** (3)	33% (1/3)	33% (1/3)	33% (1/3)		
**Surfactant A**	**1** (23)	48% (11/23)	52% (12/23)	0% (0/23)	0.095	0.19
	**Mixed** (10)	10% (1/10	90% (9/10)	0% (0/10)		
	**2** (10)	50% (5/10)	50% (5/10)	0% (0/10)		
	**3** (3)	100% (3/3)	0% (0/3)	0% (0/3)		
**TTF-1**	**1** (23)	0% (0/23)	0% (0/23)	100% (23/23)	-	-
	**Mixed** (10)	0% (0/10)	0% (0/10)	100% (10/10)		
	**2** (10)	0% (0/10)	0% (0/10)	100% (10/10)		
	**3** (3)	0% (0/3)	0% (0/3)	100% (3/3)		
**p40**	**1** (23)	0% (0/23)	9% (2/23)	91% (21/23)	0.005	0.03
	**Mixed** (10)	0% 0/10)	40% (4/10)	60% (6/10)		
	**2** (10)	0% (0/10)	80% (8/10)	20% (2/10)		
	**3** (3)	0% (0/3)	100% (3/3)*	0% (0/3)		
**CK5/6**	**1** (23)	0% (0/23)	57% (13/23)	43% (10/23	0.017	0.051
	**Mixed** (10)	10% (1/10)	80% (8/10)	10% (1/10)		
	**2** (10)	40% (4/10)	60% (6/10)	0% (0/10)		
	**3** (3)	100% (3/3)	0% (0/3)	0% (0/3)		
**CK7**	**1** (23)	0% (0/23)	0% (0/23)	100% (23/23)	-	-
	**Mixed** (10)	0% (0/10)	0% (0/10)	100% (10/10)		
	**2** (10)	0% (0/10)	0% (0/10)	100% (10/10)		
	**3** (3)	0% (0/3)	0% (0/3)	100% (3/3)		
**CK20**	**1** (23)	52% (12/23)	17% (4/23)	30% (7/23)	0.29	0.35
	**Mixed** (10)	30% (3/10)	50% (5/10)	20% (2/10)		
	**2** (10)	70% (7/10)	10% (1/10)	20% (2/10)		
	**3** (3)	100% (3/3)	0% (0/3)	0% (0/3)		

To further analyze differences between CPAM types (1, 2, mixed) statistically, Kruskal–Wallis tests were performed. A significant difference was apparent in p40 expression (*p* = 0.005; *q* = 0.03). Thereby, the Dunn’s test revealed a significant difference between type 1 and type 2 (*p* = 7.18e^−5^; *q* = 0.0002), and no significance between type 1 and mixed (*p* = 0.081; *q* = 0.081) as well as type 2 and mixed (*p* = 0.059; *q* = 0.081). The CPAM types showed a tendency to differ in CK5/6 (*p* = 0.017; *q* = 0.051). Albeit, these results were not statistically significant after correcting for multiple testing. A consequently performed pairwise Dunn’s test showed a higher frequency of CK5/6 in CPAM type 1 compared to CPAM type 2 (*p* = 0.0003; *q* = 0.0009). No differences were shown in CK5/6 between type 1 and mixed (*p* = 0.051; *q* = 0.076), as well as between type 2 and mixed (*p* = 0.16; *q* = 0.16). In addition, no statistically significant differences were observed for Napsin A (*p* = 0.37; *q* = 0.37), Surfactant A (*p* = 0.095; *q* = 0.19), and CK20 (*p* = 0.29; *q* = 0.35).

### Next-generation sequencing

As 17% (8/46) of all samples showed MCCs, comparative sequencing was performed within two different areas (mucinous and non-mucinous). Thus, NGS of 54 different regions was performed. Mutations were detected in 24% (11/46) of all samples. The frequencies were 35% (8/23) within type 1, 0% (0/10) within mixed types, 0% (0/10) within type 2, and 100% (3/3) in type 3. The following genes were mutated: *KRAS* (Exon 2), *TP53* (Exon 4), *FGFR2* (Exon 9)*.* Besides the classical *KRAS* G12D (type 1: *n* = 5) and *KRAS* G12 V mutations (type 1: *n* = 2), a *KRAS* G12D + V double mutation (type 1: *n* = 1) and a *TP53*/*KRAS* G12D co-mutation were identified (type 3: *n* = 1). The remaining two type 3 lesions both carried the same *FGFR2* C382R mutation. All mutations showed comparable allele frequencies within the mucinous and non-mucinous areas. In three mutated cases (*KRAS*: *n* = 1*, FGFR2: n* = 2), no MCCs were found, even after multiple slice sectioning. All 11 cases with mutations were resected within the first 10 months of life. For further details, see Table 4. By means of RNA-based NGS, no pathogenic gene fusions could be detected.

**Table 4:** Comparison of different mutations in mucinous cell clusters (MCCs) and non-mucinous cystic areas with regard to allele frequencies (AF)

Case-ID	CPAM type	Mutation in MCCs	AF in MCCs	Mutation in non-mucinous cystic areas	AF in non-mucinous cystic areas	Age at operation
1	1	*KRAS* G12 V	24.1%	*KRAS* G12 V	27.3%	4 days
5	1	*KRAS* G12D + *KRAS* G12 V	6.0% 20.4%	*KRAS* G12D + *KRAS* G12 V	6.7% 21.8%	11 days
6	1	*KRAS* G12D	18.7%	*KRAS* G12D	22.7%	10 month
7	1	*KRAS* G12D	24.9%	*KRAS* G12D	21.7%	1.5 month
8	1	*KRAS* G12D	29.4%	*KRAS* G12D	27.6%	13 days
9	1	*KRAS* G12D	31.5%	*KRAS* G12D	34.9%	15 days
10	1	*KRAS* G12D	21.1%	*KRAS* G12D	24.1%	3 days
22	1	no MCCs	/	*KRAS* G12 V	21.2%	12 days
41	3	*KRAS* G12D + *TP53* C124 Ter	20.4% 4.3%	*KRAS* G12D + *TP53* C124 Ter	6.7% 7.7%	4 days
42	3	No MCCs	/	*FGFR2* C382R	29.3%	3 days
43	3	No MCCs	/	*FGFR2* C382R	32.2%	6 days

### Comparison of KRAS-mutated CPAMs regarding conventional morphology, immunohistochemistry, and molecular data

Of the nine *KRAS*-mutated cases, 56% (5/9) had a cyst size > 2 cm and 34% (3/9) ≤ 2 cm. The remaining two cases (type 3) showed a sponge-like cystic formation with a diameter up to 4.5 cm. A total of 100% (9/9) were positive for napsin A, TTF-1, CK7, and p40 within the epithelia of the cyst. CK5/6 positivity was observed in 89% (8/9), CK20 in 44% (4/9), and surfactant A in 33% (3/9). Differences between *KRAS*-mutated and *KRAS* wild-type CPAMs were further analyzed using Mann–Whitney *U* tests. Statistical analyses revealed that *KRAS*-mutated samples were significantly more often positive for napsin A compared to *KRAS* wild-type samples (*p* = 0.0005; *q* = 0.003). For surfactant A (*p* = 0.085; *q* = 0.22), CK20 (*p* = 0.11; *q* = 0.22), p40 (*p* = 0.42; *q* = 0.5), CK5/6 (*p* = 0.51; *q* = 0.51), and cyst size (*p* = 0.2; *q* = 0.3), no statistically significant differences were found.

## Discussion

To the best of our knowledge, this study represents the most comprehensive analysis of CPAM types 1–3 to date, integrating morphological, immunohistochemical, and molecular data. Our immunohistochemical analyses aimed to delineate protein expression profiles across different pulmonary regions (central vs. peripheral), while NGS was employed to identify potential molecular drivers. Notably, we sought to validate prior findings suggesting that KRAS mutations are not restricted to mucinous cell clusters (MCCs), but may also be present in surrounding non-mucinous epithelium.

### Conventional morphology and immunohistochemistry

Type 1 CPAM is widely regarded as the most prevalent subtype, comprising 60–70% of cases, followed by type 2 (15–20%) and type 3 (5–10%) [6, 14, 17, 25, 26]. Our findings (50% type 1, 22% type 2, and 6% type 3) align with this distribution, though variations have been reported [12, 27], with some studies identifying type 2 as the most frequent subtype [8, 9, 28]. Such discrepancies may be attributed to enhanced prenatal detection of asymptomatic lesions [9].

Stocker’s original 1977 classification [5] was expanded in 2002 to include five subtypes, primarily based on cyst size and morphology [6]. Conventionally, type 1 lesions are characterized by cysts > 2 cm, whereas type 2 lesions contain smaller cysts (≤ 2 cm), and type 3 lesions exhibit sponge-like microcystic areas [1, 5]. However, emerging evidence suggests that cyst size alone may not reliably predict histological subtypes in up to 18% of cases [9, 18, 23], a finding corroborated and even exceeded in our study (Table 2). This may be partially explained by the fact that 36% of the cysts in our cohort measured near the 2 cm threshold (Supplementary Table 1). Previous studies [9, 12] have emphasized that cyst size may be influenced by factors such as inflation and infection, leading to proposals for standardized histopathological assessment protocols (see supplementary data in [12]).

Beyond classical CPAM subtypes, hybrid lesions (CPAM with sequestration) [28] and mixed CPAM types have increasingly been recognized. The latter may occasionally show overlapping features of type 1 + 3 [23] or even type 1 + 4 [29], but typically involve type 1 + 2 [4, 13, 30] (most common, 22% in our cohort). This suggests that CPAM lesions may represent a developmental continuum, reflecting disruptions at different stages of lung morphogenesis, an idea further supported by our protein expression patterns.

Immunohistochemical analysis revealed strong TTF-1 and CK7 positivity across all CPAM subtypes, consistent with their fundamental roles in pulmonary epithelial differentiation. TTF-1 plays a crucial role in embryonic and fetal lung development [31], with expression persisting in type 2 pneumocytes during later gestation [7]. The strong TTF-1 positivity observed in types 1–3 (both in our data and in the literature [7, 12, 14, 22]) mirrors expression patterns typically found in the fetal lung during the pseudoglandular phase [7], indicating that the underlying insult likely occurs between the 5th and 17th weeks of gestation [25]. Meanwhile, CK5/6, a marker of basal epithelial cells, emerges by the 14 th week of gestation [32]. Our study is among the first to report p40 and CK5/6 expression in CPAM. The predominance of p40 and CK5/6 expression in type 1 compared to type 2 CPAMs suggests that earlier developmental disruptions may preferentially affect more centrally located lung structures, as by analogy to, e.g., p63, these markers are typically enriched in central airways but diminish in peripheral alveolar regions [33]. Napsin A, an asparagine protease, is routinely used in conjunction with TTF-1 for the diagnosis of pulmonary carcinomas and metastases [34]. Its expression begins around the 12 th week of gestation in fetal pulmonary glandular structures and is thought to decline to adult-like levels by around the 30 th week [35]. To date, only two case reports have documented Napsin A expression in CPAM. Hasegawa et al. [36] described combined Napsin A and TTF-1 positivity in a CPAM type 1 associated with adenocarcinoma in an 80-year-old patient, while Koopman et al. [22] reported Napsin A-positive papillary epithelial cells in a type 1 lesion of a 6-week-old infant. Partial expression of Surfactant A in CPAM types 1–3 is known from the 12 th week of gestation [25]. In our cohort, approximately 60% showed partial and discontinuous expression of both Napsin A and Surfactant A. As with CK5/6 and p40, these diverse expression patterns likely reflect varying cellular compositions at distinct phases of lung development.

The role of MCCs as potential precursors of malignancy has been a topic of debate. MCCs are most frequently associated with type 1 CPAM, with reported prevalence ranging from 32 to 83% [5, 9, 26]. In their recent larger study, Nelson et al. [23] stated: “we have never seen mucinous cells in classic type 2 cases.” However, MCCs have also been documented in type 2 [10, 12, 21, 23], type 3 [10, 16, 23], and mixed types [23, 37]. In our cohort, no MCCs or molecular alterations (see below) were detected in either type 2 or mixed-type CPAMs. Given that type 1 and type 2 components may co-occur within the same lesion, previously reported MCCs in type 2 CPAMs could potentially reflect sampling errors in cases of mixed-type morphology. This observation may further support the continuum model hypothesis, which proposes a morphological and possibly molecular spectrum rather than strictly distinct CPAM subtypes. One large study of 671 MCCs noted multi-focal distribution in 82% of cases, most often with extracystic growth, followed by combined extra and intracystic involvement, and lastly, purely intracystic growth [16]. Consistent with prior studies [16, 17], MCCs in our series appeared in 17% of all samples (30% in type 1; 33% in type 3). A total of 75% displayed multi-focal distribution, combining extra- and intracystic localization, with a predominance of extracystic growth. Only one sample exhibited an exclusively unifocal, purely intracystic MCC. Because such small clusters can be overlooked on H&E staining alone, adding PAS-staining to routine protocols is advisable.

### Next-generation sequencing

*KRAS* functions as a molecular switch in signal transduction, playing a critical role in regulating cellular growth and differentiation. Mutations in *KRAS* are among the most frequently observed alterations in human cancers [38] and are well-documented in MCCs [12, 15, 20, 23].

These mutations are considered potential drivers of malignant transformation, including progression to metastatic mucinous lung adenocarcinoma [10, 12, 14, 17, 19, 21]. Notably, some studies have based the diagnosis of carcinoma solely on the detection of *KRAS* mutations within MCCs [19, 21]. This raises the question of whether adjacent non-mucinous congenital pulmonary airway malformation tissue also harbors such mutations. While Fakler et al. [10] reported an absence of *KRAS* mutations in these adjacent regions, our findings align with recent studies [12, 22, 23], demonstrating that identical mutations are present in the surrounding non-mucinous epithelium (with comparable allele frequencies). This reinforces the principle that malignancy diagnoses should not be based solely on the detection of a mutation. Analogies can be drawn to *BRAF* mutations in melanocytic nevi [39] and *KRAS* mutations in precursor lesions of pancreatic cancer [40]. Additionally, *KRAS* mutations have been identified in CPAMs without MCCs [12, 23], as observed in one of our samples.

Nelson et al. [16] evaluated the malignant potential of MCCs, comparing them to adult adenocarcinomas. A clear histological distinction between the two entities was challenging due to similarities in nuclear features and size. Moreover, MCCs and the surrounding epithelium exhibited a significantly higher proliferation index compared to adenocarcinomas. Interestingly, healthy infant lung tissue displayed an average proliferation rate approximately 2.5 times higher than adult lung tissue [16]. These findings suggest that diagnosing carcinoma in these contexts requires considerable caution. Some authors have hypothesized that *KRAS*-mutated MCCs, despite their morpho-molecular resemblance to mucinous adenocarcinomas, may generally exhibit benign behavior [16, 18]. Reports of malignant potential, including distant metastases and fatal outcomes, have often been attributed to incomplete surgical resection or delayed intervention [19, 41]. However, no recurrences or metastases have been reported following complete surgical resection [23, 26]. Due to the lack of long-term data on asymptomatic, unresected CPAMs, the risk of malignant transformation in untreated lesions remains uncertain. Conversely, Nelson et al. [9] demonstrated that mucinous cell clusters (MCCs) in infantile CPAMs histologically resemble adult mucinous adenocarcinoma, yet are not associated with adverse outcomes when completely resected. In these cases, follow-up imaging within the first year after surgery showed no signs of recurrence or metastatic spread—independent of *KRAS* mutation status [9, 19]. To date, no consensus or official guidelines exist regarding post-operative surveillance protocols. Future studies should focus on gathering systematic follow-up data, particularly in mutation-driven CPAMs, to improve understanding of their clinical behavior and potential risks.

One might argue, by analogy to adult cancers, that additional mutations beyond *KRAS* may be required for tumorigenesis. Although most studies on CPAM-associated adenocarcinoma have identified single *KRAS* mutations [10, 15, 17, 20, 21], recent evidence has revealed *KRAS/GNAS* co-mutations [17, 18]. In our study, we identified one case with a *KRAS* double mutation (G12D/G12 V) and another with a *KRAS–TP53* co-mutation. To the best of our knowledge, this has not been previously reported in CPAMs, and especially *KRAS* double mutations are particularly rare in carcinomas overall [42]. In contrast, *KRAS–TP53* co-mutations are more frequently observed and are often associated with poor prognosis in cancers [43]. The co-occurrence of *TP53* alterations aligns with classical lung cancer biology, suggesting a potentially higher risk of malignant transformation in such cases [44]. However, in our study, early surgical intervention and subsequent follow-up did not reveal malignant progression, leaving this hypothesis speculative. Interestingly, our statistical analyses revealed that *KRAS*-mutated samples were significantly more often positive for Napsin A compared to *KRAS* wild-type samples. We suggest that this is a phenomenon by chance; however, further research is needed to gain insight at the cellular level.

Additionally, we identified *FGFR2* mutations in two of three type 3 CPAM samples. So far, this mutation has only been anecdotally reported in a mixed type 1 + 3 CPAM [23]. Furthermore, strong *FGFR2* expression has been observed in cystic epithelium, MCCs of CPAM types 1–3, and normal lung tissue [10, 45]. *FGFR2*, a tyrosine kinase receptor, plays a critical role in early lung development, with disruptions potentially leading to respiratory anomalies and tumorigenesis [46]. As 24% of our samples showed mutations, the question arises whether these lesions might represent a distinct subtype. Further research is needed to identify the (stem) cell of origin (e.g., by single-cell sequencing) and to gain insight into first steps of pathogenesis.

This study has several limitations. Due to its retrospective design, potential biases in data collection and documentation cannot be excluded. Although the case number is relatively high for this rare entity and the study was conducted across multiple centers, the total number of included cases (*n* = 46) remains limited. Moreover, long-term clinical follow-up data were not available, restricting conclusions regarding the prognostic relevance of *KRAS* and/or *FGFR2* mutations. In two cases, cyst size could not be assessed due to incomplete histopathological records, limiting the morphological classification. The small cohort size also reduced the statistical power for subgroup analyses; in particular, CPAM type 3 could not be evaluated separately due to the low number of cases. The absence of mucinous cell clusters (MCCs) in type 2 and mixed lesions may likewise reflect the limited sample size (*n* = 10). It is noteworthy that all 11 cases with mutations were resected within the first 10 months of life. Nonetheless, we do not consider this to reflect a systematic age-related bias, as the vast majority of patients (97.8%, 45/46) were nine years old or younger at the time of surgery, and still 76.1% (35/46) were within the first year of life (see Supplementary Table 1).

## Conclusions

A clear-cut assignment to CPAM classification may be challenging in a subset of samples, as patterns favoring type 1 and/or type 2 might be seen within a single lesion. However, the occurrence of these mixed types supports the hypothesis that such lesions rather represent a continuum of developmental disturbances occurring at different stages of lung development and, consequently, at different localizations within the bronchioloalveolar tree. This is further corroborated by the divergent protein expression patterns observed in the cystic epithelium. Our findings confirm that identical *KRAS* mutations can be present in MCCs and adjacent non-mucinous CPAM tissue, underscoring the need to avoid diagnosing carcinoma based solely on mutation analysis. With advances in parallel sequencing, additional mutations—such as *FGFR2* or double mutations—are likely to be identified. Given that one-quarter of our samples exhibited molecular alterations, a mutation-driven CPAM subtype, particularly within types 1 and 3, may warrant further investigation.

## Supplementary Information

Below is the link to the electronic supplementary material. 

ESM1 (DOCX 286 KB)
